# Cardiac Morpho-Functional Changes, Inflammation and Fibrosis in Systemic Sclerosis—A Pilot Study of a Tertiary Center Cohort

**DOI:** 10.3390/diagnostics15030393

**Published:** 2025-02-06

**Authors:** Karolina Dorniak, Zuzanna Gogulska, Alessandro Viti, Anna Glińska, Dorota Kulawiak-Gałąska, Jadwiga Fijałkowska, Anna Wojteczek, Dagmara Wojtowicz, Katarzyna Sienkiewicz, Marcin Hellmann, Żaneta Smoleńska

**Affiliations:** 1 Department of Cardiac Diagnostics, Faculty of Medicine, Medical University of Gdańsk, 80-210 Gdańsk, Poland; 2 Department of Internal Medicine, Connective Tissue Diseases & Geriatrics, Medical University of Gdańsk, 80-210 Gdańsk, Poland; 3 Faculty of Medicine, University of Naples Federico II, 80138 Naples, Italy; 4 2nd Department of Radiology, Faculty of Health Sciences, Medical University of Gdańsk, 80-210 Gdańsk, Poland; 5 Department of Radiology, Faculty of Medicine, Medical University of Gdańsk, 80-210 Gdańsk, Poland

**Keywords:** systemic sclerosis, heart involvement, cardiac magnetic resonance

## Abstract

**Background**: Cardiac involvement (CI) in systemic sclerosis (SSc) is frequently subclinical and it can be identified in up to 80% of autopsied hearts. If present, symptoms are related to adverse prognosis, and CI represents one of the predominant causes of SSc-related mortality. **Methods**: A total of 20 patients with a diagnosis of SSc were included and followed up, and 37 volunteers were included and subsequently scanned on a 1.5T MR system. **Results**: Overall, thirteen (65%) patients had one or more abnormal cardiac findings in CMR (defined as CI[+]), of which in seven (35%), baseline ECGs and standard echocardiograms were normal or unspecific. Compared to healthy volunteers, SSc patients had a lower LVEF% (56.6% vs. 61.6%; *p* = 0.0131), longer T1 (1028.3 ms vs. 993.1 ms; *p* = 0.0049) and T2 relaxation times (48.24 ms vs. 43 ms *p* = 0.0011), and higher extracellular volume (ECV, 27.9% vs. 26.0%; *p* = 0.0112). However, no difference in CMR-derived, feature-tracking GLS values between patients and healthy controls was found (−15.5[2,8] vs. −16.3[1,1], respectively, *p* = 0.11). Over 3.4 (1.9–5.5) years, three patients (15%) died, and two others (10%) sustained major cardiac complications. **Conclusions**: Cardiac magnetic resonance with modern quantitative techniques reveals subtle morpho-functional alterations and thus allows for early diagnosis of myocardial involvement in systemic sclerosis. Our findings emphasize the need for extended diagnostic workup in these patients and demonstrate the ability of cardiac MR to select patients requiring closer follow-up and/or treatment decisions.

## 1. Introduction

Systemic sclerosis (SSc) is a rare connective tissue disease characterized by autoimmunity, vasculopathy, and fibrosis of the skin and internal organs [1]. This condition often results in disability and premature death, primarily due to organ complications such as lung fibrosis, kidney disease, pulmonary arterial hypertension (PAH), and cardiac involvement (CI) [2,3]. To date, no curative treatment has been identified. The available therapies only slow the progression of SSc and often have significant side effects. The clinical manifestations of SSc vary widely, and tools for predicting disease progression or risk of organ involvement are still a matter of debate, especially with regard to CI [4,5]. Two primary clinical subgroups: diffuse SSc (dcSSc) and limited SSc (lcSSc) were identified based on skin and organ involvement profiles [6]. The lcSSc subtype is characterized by localized skin fibrosis (affecting the face and distal parts of extremities) and milder organ involvement, except for PAH. Notably, Raynaud’s phenomenon (RP) often precedes other symptoms in this group by several years. Conversely, dcSSc is defined by extensive skin involvement and a more aggressive progression of organ complications.

Racial and gender differences in SSc clinical presentation and outcomes are well documented [7,8]. Although certain autoantibodies, such as anti-topoisomerase I (anti-Scl-70), anti-centromere, and anti-polymerase III (anti-RNPIII), are commonly used for SSc diagnosis, they are not effective in screening for organ involvement. However, some studies have suggested that antibodies such as anti-Scl-70, anti-RNPIII, anti-Ku, anti-U3RNP, and anti-histone are linked to an increased risk of SSc-related cardiac disease [9]. A more recent study of a fairly large cohort of SSc patients showed that the presence of an array of SSc-related autoantibodies does not closely correspond to specific clinical features of SSc and various types of SSc overlap syndromes [10]. Recently, however, new organ-specific, anti-heart, and anti-intercalated disk autoantibodies were identified as fairly specific markers of cardiac involvement in SSc [11], having been linked to clinical presentation as well as prognosis.

Cardiac involvement in SSc is frequently subclinical, and symptomatic heart disease is reported in up to 35% of patients [4,5,12,13]. If present, symptoms are non-specific and may be difficult to attribute directly to SSc-related heart disease. Moreover, CI can be identified in up to 80% of autopsied hearts of patients with SSc, affecting the myocardium, pericardium, endocardium, and the coronaries [14]. The underlying pathology of this condition is multifaceted. Cardiac tissue damage is thought to primarily result from the cardiac Raynaud’s phenomenon (RP), which leads to recurrent ischemia–reperfusion injuries that can be linked to microvasculopathy, inflammation, and fibrosis [2,3]. Furthermore, secondary CI (stemming from interstitial lung disease [ILD], kidney failure, or cardiovascular events) is also a significant concern [3,15]. Heart involvement alone may cause up to 27% of SSc-related deaths and is one of the leading causes of mortality [15]. Cardiac death usually results from life-threatening arrhythmias, heart failure, or sudden cardiac arrest. Diagnostic tools that can be used for heart involvement in SSc include electrocardiography (ECG), echocardiography, serum markers (troponin, BNP), endomyocardial biopsy, and magnetic resonance imaging [16]. Cardiac magnetic resonance (CMR) with advanced tissue characterization (including parametric mapping of the T1 and T2 relaxation times and extracellular volume [ECV] fraction of the myocardium) provides a unique non-invasive insight into early (inflammation, edema) and late (fibrosis, necrosis) stages of primary myocardial involvement in those patients. Its added value over standard transthoracic echocardiography is well documented and hence it has recently been recommended for early detection of subclinical CI in SSc [16,17,18].

In this study, we aimed to emphasize the necessity of searching for CI in SSc and illustrate the usefulness of contemporary cardiac magnetic resonance imaging techniques for early diagnosis. We conducted a preliminary, single-center CMR study of the consecutive SSc patients and compared them to a healthy control group. CMR results were analyzed along with clinical presentations, standard diagnostic tests, and follow-up data as a preliminary study of our tertiary center cohort. 

## 2. Patients and Methods

Twenty consecutive patients with systemic sclerosis by the ACR/EULAR (2013) criteria [19] (51 ± 14 years, 14 [70%] female) referred to a tertiary center, with any nonspecific symptoms and/or signs suggestive of possible heart involvement, including arrhythmia/palpitation, peripheral edema, chest pain, or exercise intolerance, were prospectively included. Routine clinical assessment was performed at inclusion for all patients, including skin and organ involvement and disease activity defined as an EUSTAR score ≥ 2.5 [20]. Laboratory tests included liver and kidney function, lipid profile, BNP or NT-pro BNP, and extended antibody panel. All patients underwent standard 12-lead ECG, 24-hour ECG monitoring, standard transthoracic echocardiography, and CMR. Cardiac MR exams were performed on a 1.5 Tesla scanner (Magnetom Area or Magnetom Sola, Siemens AG, Erlangen, Germany) using a standardized imaging protocol. Scout acquisitions were followed by three long-axis cines and a short-axis cine stack for morpho-functional assessment. Cardiac parametric mapping sequences included longitudinal (T1) and transverse (T2) relaxation time measurements (MOLLI [Modified Look-Locker] sequence for T1- and a T2-prepared bSSFP sequence for T2-mapping, respectively [MyoMaps, Siemens Healthineers, Erlangen, Germany]). Late gadolinium enhancement (LGE) with phase-sensitive inversion recovery (PSIR) was acquired both in the long axes and a short axis stack, within 7–15 min after injection of 0.1 mmol/kg of gadobutrol (Gadovist, Bayer AG, Leverkusen, Germany). CMR images were evaluated by two experienced imagers (a cardiologist and a radiologist) using proprietary commercial software (SyngoVia VB40, Siemens Healthineers, Erlangen, Germany). Global longitudinal strain (GLS) was then calculated in patients and controls, using a feature-tracking strain measurement technique based on standard cine CMR series and the Segment CMR feature-tracking strain module of the commercially available Segment CMR software v.4.0 (Medviso AB, Lund, Sweden). The study was approved by the institutional ethics committee (No NKBBN/57/2021) and all patients provided written informed consent. Statistical analysis was carried out as follows. For categorical variables, descriptive statistics consisted of absolute and relative frequencies while continuous variables were reported as the mean and standard deviation (SD) or median (Q1, Q3) as appropriate. The Fisher test was used to compare categorical variables, and a two-tailed unpaired *t*-test was used for the comparison of continuous variables. Welch’s correction was performed. *p*-values of <0.05 were considered significant.

## 3. Results

The mean time from the first symptoms in the study group was 9.9 (6,8) years and the mean time from SSc diagnosis by a rheumatologist was 7.0 (6,7) years. Median follow-up duration was 3.4 (1.9–5.5) years. Data on clinical presentations, medical history, baseline laboratory tests, lung function tests, disease type, and EUSTAR activity score at inclusion are summarized in Table 1. CMR findings were compared to a group of healthy controls (*n* = 37; Table 2). Systemic sclerosis patients had significantly lower LVEF% (56.6% vs. 61.6%; *p* = 0.0131), higher left ventricular end-systolic volume index (LVESVI, 38.9 mL/m^2^ vs. 31.6 mL/m^2^; *p* = 0.0113), longer T1 (1028.3 ms vs. 993.1 ms; *p* = 0.0049) and T2 relaxation times (48.24 ms vs. 43 ms *p* = 0.0011), and higher extracellular volume (ECV, 27.9% vs. 26.0%; *p* = 0.0112) compared to healthy controls. No difference in CMR-derived, feature-tracking GLS values between patients and healthy controls was found (−15.5[2,8] vs. −16.3[1,1], respectively, *p* = 0.11).

**Figure 1:**
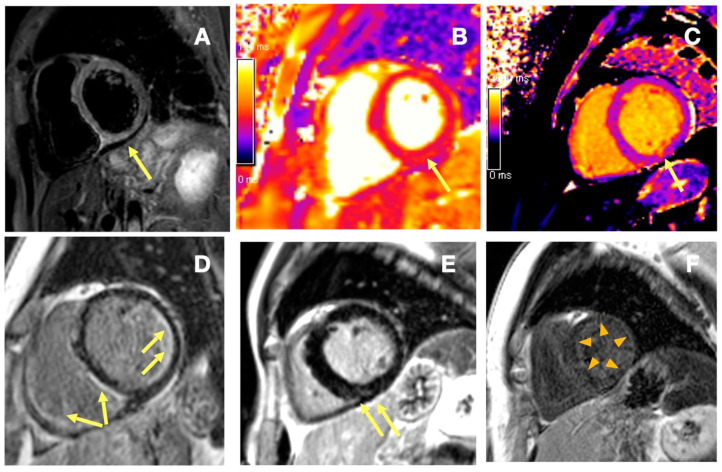
(**A–C**) Sixty-six-year-old woman with a recent (6 months prior) DSSc diagnosis, normal transthoracic echocardiography, borderline LV size, and normal function on CMR (LVEDVI of 99 mL/m^2^ [ref. range 61–95 mL/m^2^] and LVEF of 65%, GLS of −17.4% (within the reference range)), normal standard ECG and paroxysmal second degree AV block. (**A**) T2-weighted images (T2STIR) showed high signal within the basal segments of the inferior and inferoseptal wall (ratio of myocardial to skeletal muscle signal >2), matched by locally increased T2 relaxation time of 55 ms in the inferior wall. The institutional reference range for T2 time was 39–49 ms ((**B**), arrow). Images (**A**,**B**) are consistent with localized myocardial edema. In (**C**), locally increased native T1 relaxation time (1081 ms) can be noted (the institutional reference range 953–1035 ms), corresponding both to edema and to subtle areas of disperse subendocardial late gadolinium enhancement in the inferior wall. (**D**–**F**) Patterns of inflammatory injury in SSc patients, late gadolinium enhancement images. (**D**) Fourty-one year-old female patient with DSSc, with generalized subendocardial late gadolinium enhancement (arrows), suggestive of the presence of a degree of irreversible injury in the subendocardium. (**E**) Fifty-nine-year-old male SSc patient with overt heart involvement, elevated BNP, enlarged ventricles and reduced LVEF of 41%. Nonischemic intramyocardial late gadolinium enhancement in the basal inferior wall in the short axis. (**F**) Sixty-five-year-old female patient 2 yrs from DSSc diagnosis, with normal echocardiography, and normal ECG; subtle dispersed generalized late gadolinium enhancement can be noted (arrowheads). (Siemens Aera 1.5T, Erlangen, Germany). AV—atrio-ventricular, BNP—brain natriuretic peptide, CMR—cardiac magnetic resonance, GLS—global longitudinal strain, LVEDVI—left ventricular end-diastolic volume index, LVEF—left ventricular ejection fraction, T2STIR—T2-weighted short-tau inversion recovery sequence.

Overall, thirteen (65%) patients had one or more abnormal cardiac findings in CMR (CI[+]), of which for seven (35%), baseline ECGs and standard echocardiograms were normal or borderline normal (Figure 1). Active disease (i.e., EUSTAR activity score ≥ 2.5) was noted in the majority (eight of thirteen) of the CI[+] patients, whereas only one of seven CI[−] patients had active disease. No specific autoantibodies (including anti-topoisomerase I [anti-Scl70], anti-Ku, anti-U3 RNP, anti-histone, and anti-RNA polymerase I, II, and III) were found to be more prevalent in our patients with CI. Cardiac involvement was more frequent among DSSc patients (eleven of thirteen), with greater systolic dysfunction and more pronounced tissue alterations than in LSSc patients (two of seven).

Of the four patients with abnormal RV findings (all four CI[+] by definition), ILD or PAH was present in three. Conversely, 13 of 16 patients with ILD/PAH had no RV enlargement and/or dysfunction by CMR.

During a median follow-up of 3.4 (1.9–5.5) years, three patients in our group (15%) died (heart failure—one; gastrointestinal complications—one; breast cancer—one). One patient required permanent pacemaker implantation for second-degree AV block as a consequence of cardiac involvement. One patient with DSSc developed aggressive, treatment-refractory disease with multiple unplanned hospitalizations for a variety of complications including extensive myocardial involvement and cardiac tamponade followed by constriction. Overall, during follow-up, treatment escalation and/or change were required in thirteen patients; in five patients, the treatment was reduced or remained unchanged during follow-up. No change in treatment was noted in two patients (10%) who were lost to follow up after one year and two years from inclusion, respectively.

## 4. Discussion

This study demonstrates how a multiparametric approach with CMR improves the detection of early cardiac involvement in systemic sclerosis. Compared with a group of healthy controls, our patients had significantly lower LVEF, higher LVESVI, longer T1 and T2 relaxation times, and higher ECV as compared to controls. This, according to a recent review [18] represents unprecedented and so far unmatched diagnostic insight into the disease process. Indeed, our results are largely in concordance with the previous report, including a recent study by Gargani et al. [21], who evaluated the added value of CMR in the diagnostic workup of a large group of SSc patients. We found slightly higher rates of LGE (50% vs. 28%) and slightly more prevalent edema in our group (5% vs. 2.5%) both likely related to the fact that in the study by Gargani, unlike our group, unselected patients with SSc were included, irrespective of the presence of symptoms suggestive of possible cardiac involvement [21]. A number of studies pointed to the role of CMR in the diagnosis of CI in a variety of SSc cohorts, uniformly confirming the added value of CMR-derived information in the decision-making process. Our results refer to the less well-studied central European population, which, given the racial and ethnic differences in the disease spectrum, [7] provides additional rationale for conducting similar studies in different patient cohorts.

Contrary to previously reported GLS alterations by speckle tracking echocardiography in SSc patients, where global longitudinal strain values were significantly impaired [22], in our group, no difference in CMR-derived feature-tracking GLS values between patients and healthy controls was found, which further emphasizes the need for the multiparametric approach offered by CMR in these patients. Interestingly, contrary to a study by Pereira et al. [9], where anti-topoisomerase I autoantibodies were reported as more common in CI[+] SSc patients, no specific autoantibodies (including anti-topoisomerase I [anti-Scl70], anti-Ku, anti-U3 RNP, anti-histone, and anti-RNA polymerase I, II and III) were found to be more prevalent in our patients with CI. This can be at least in part due to a relatively small number of patients in our group, but it is also in line with a large Italian study by Ferri et al. [23], in which no specific antibody type could be identified in those patients as more common compared to CI[−]. Cardiac involvement was more frequent among DSSc patients (eleven of thirteen), with greater systolic dysfunction and more pronounced tissue alterations than in LSSc patients (two of seven). Other authors reported similar proportions of CI in DSSC and LSSC patients [24]. It is of note that CMR allowed for the detection of cardiac tissue damage (including edema/abnormal T1/T2/ECV and/or non-ischemic fibrosis) in three (15%) of the patients whose ECGs and echocardiograms were normal, as well as added new tissue information in four (20%) others, in whom borderline nonspecific echo abnormalities were detected. It is well known that routine cardiac workups may not allow for the detection of subtle cardiovascular alterations. Indeed, morpho-functional and laboratory test results may remain largely unchanged despite ongoing heart involvement, and clinically overt heart involvement is a generally late and variable phenomenon, ranging from 10 to 44%, depending primarily on the assessment criteria [25]. This may contribute to a higher risk of mortality once the cardiac involvement becomes clinically overt, as exemplified by a meta-analysis of cohort studies by Ioannidis et al. where mortality rates were greater in patients with cardiac involvement compared to those with renal or pulmonary SSc-related disease [26].

RV alterations found in patients with SSc merit special attention, as they can be either primary or secondary to pulmonary arterial or lung involvement [27]. Notably, 13 of 16 patients with ILD/PAH in our study had no RV enlargement and/or dysfunction by CMR, which is in contrast to the findings of Meune et al., who reported a high prevalence of right ventricular systolic dysfunction in early systemic sclerosis, irrespective of the presence of pulmonary arterial hypertension [27]. They concluded that RV systolic dysfunction, as determined by radionuclide ventriculography, may result from other causes, such as primary myocardial involvement. The markedly lower prevalence of RV enlargement/dysfunction in our SSc cohort could be at least partially explained by the fact that lung and pulmonary circulation involvement may affect morpho-functional RV parameters at a later stage. Methodological differences (radionuclide ventriculography vs. cardiac magnetic resonance), may also have affected these divergent results.

Three deaths (15%) were recorded over the follow-up period, one of which was due to heart failure. Two other patients sustained serious or life-threatening cardiovascular events due to CI, including pacemaker implantation and pericardial constriction. Similar mortality rates over a similar follow-up period were reported by other authors. In one of the fairly large prospective outcome studies, which included 120 SSc patients, the overall mortality rate was 19.2% over a five-year period [28]. Conversely, in a large study from the EULAR Scleroderma Trials and Research (EUSTAR) database published in 2010 that included 5280 patient-years of observation in 5620 patients, the overall mortality rate was 5.2% over a median follow-up of 0.9 years per patient [29].

Our study has several limitations. Firstly, for clarity, we did not present morpho-functional echocardiographic data, as similar information is available from CMR (which currently represents a reference method for LV volumes and function). Second, no systematic assessment of reversible ischemia was performed. However, as per clinical indications, it was performed for three patients. No reversible ischemia was detected, and no revascularization procedure was required in any of our patients during the study. Moreover, coronary microvascular alterations, which play a major role in CI pathogenesis, were not directly addressed in our study. It could also be argued that the non-ischemic LGE identified in our patients has low specificity and could potentially have resulted from viral myocarditis. However, no clinically overt episodes of viral myocarditis were noted in the medical records of our patients. Lastly, given the long time from the onset of first symptoms to diagnosis in the study group, our data from a tertiary center may not necessarily be representative of the entire SSc population.

## 5. Conclusions

Our preliminary data confirm that cardiac involvement is frequent in patients with systemic sclerosis. Contemporary cardiac magnetic resonance with quantitative tissue characterization techniques reveals subtle morpho-functional alterations and thus allows the early diagnosis of myocardial involvement, while routine cardiac diagnostic tests can be unrevealing in a significant proportion of patients. Therefore, our findings emphasize the need for extended diagnostic workups in these patients. Even though its wider availability remains limited, cardiac MR identifies patients requiring closer follow-up and/or early treatment decisions.

## Figures and Tables

**Table 1:** Study group characteristics.

Study Group Characteristics at Inclusion			
	Patients (*n* = 20)	LSSC (*n* = 7)	DSSC (*n* = 13)
Age [years]	49.9 ± 14.73	43.57 ± 21.84	54.85 ± 12.12
Sex (F) [*n*]	14	5	9
Symptoms at inclusion [y]	5.80 ± 5.75	4.57 ± 1.72	6.46 ± 6.67
Years from Raynaud’s onset to diagnosis [y]	1.85 ± 4.19	3.0 ± 4.92	1.23 ± 2.45
Disease activity index at inclusion (EUSTAR > 2.5 points)	9	2	7
ILD	16	5	11
Pulmonary hypertension	2	0	2
Heart failure	4	0	4
Chest Pain	3	0	3
Dyspnea	9	3	6
Arrhtyhmia	8	2	6
Musculoskeletal symptoms	15	4	11
CKD (eGFR < 60 mL/min/1.73 m^2^)	2	0	2
Gastrointestinal symptoms	16	5	11
Overlap syndrome *	4	3	1
Follow-Up Data			
Death	3	0	3
DIsease progression/Treatment escalation	5	2	3
Treatment unchanged	10	3	7
Lost to follow-up	2	1	1

* Overlap syndromes: rheumatoid arthritis (2), Sjogren’s (1), sarcoidosis (1).

**Table 2:** CMR findings in the study group compared with healthy volunteers.

	Patients *n* = 20	Controls *n* = 37	*p*-Value
Age [years]	49.9 ± 13.7	44.4 ± 15.4	0.215
Sex (F)	13	20	0.310
LVEF	56.6 ± 7.40	61.6 ± 4.99	**0.002**
LVEDVI	85.8 ± 13.9	81.1 ± 12.6	0.101
LVESVI	38.9 ± 10.6	31.6 ± 7.07	**0.002**
LVMI	62.6 ± 12.4	61.7 ± 9.83 *n* = 17	0.409
RVEF	53.0 ± 8.55	53.7 ± 7.07	0.367
RVEDVI	85.5 ± 17.00	84.2 ± 13.8	0.376
RVESVI	40.4 ± 12.9	40.1 ± 11.7	0.457
T1 relaxation time	1029.31 ± 44.11 *n* = 16	993 ± 21 ms	**0.0002**
T2 relaxation time	48.13 ± 2.90 *n* = 16	44 ± 2.4 ms	**0.0001**
ECV	27.93 ± 2.60 *n* = 15	26 ± 3%	**0.0341**

## Data Availability

Research data supporting the reported results are available from the corresponding author upon reasonable request.
